# Chagasic heart failure in a pregnant woman in a non-endemic area: case report and long-term follow-up

**DOI:** 10.1515/crpm-2021-0074

**Published:** 2022-08-19

**Authors:** Teresa Gastañaga-Holguera, Virginia González-González, Paloma Merino-Amador

**Affiliations:** Obstetrics and Gynecology Department, Hospital Clínico San Carlos, Madrid, Spain; Microbiology Department, Hospital Clínico San Carlos, Madrid, Spain

**Keywords:** Chagas disease and congenital transmission, Chagasic cardiac disease, chronic Chagas disease, pregnancy and Chagas disease, pregnancy and Chagasic heart failure

## Abstract

**Objectives:**

Chagas disease (CD) is caused by infection with the protozoan *Trypanosoma cruzi*, a parasite that nests in various tissues, causing irreversible cardiac damage in 30% of patients with chronic disease and neurological or digestive lesions in 10%. CD is now found in areas receiving migrant populations where no vector-borne transmission occurs. Chagasic cardiomyopathy (CC) is the most serious complication of the chronic phase of CD and the major cause of morbidity and mortality among patients with CD.

**Case presentation:**

Bolivian woman at 38 weeks of gestation was admitted at the emergency room with the diagnosis of congestive heart failure. Cesarean section was performed and maternal hypotension and uterine atony occurred. Dilated myocardiopathy with severe left ventricle dysfunction was diagnosed. The patient referred positive serology for *T. cruzi* and polymerase chain reaction (PCR) was positive so benznidazole therapy was started. She was discharged due to progressive improvement with cardiological treatment and implantable cardioverter defibrillator was placed 5 years later for the prevention of sudden cardiac death.

**Conclusions:**

The diagnosis of CC in non-endemic areas requires a high index of suspicion and it is based on serology. Antiparasitic drugs are almost 100% effective in infected newborn babies and highly effective in the treatment of patients in the acute stage of the disease. However, the efficacy of both drugs decreases the longer a person has been infected. Treatment of CC that causes chronic heart failure is similar to that in non-Chagasic etiology.

## Introduction

Chagas disease (CD) or American trypanosomiasis is a systemic chronic parasitic infection caused by *Trypanosoma cruzi* (*T. cruzi*) and transmitted by Triatomine insect vectors in endemic areas. Chagas disease is endemic in 21 countries throughout Mexico, Central and South America. CD primarily affects low-income populations and is a major cause of heart disease and cardiovascular-related deaths in areas where it is endemic. Infection can be also acquired by blood transfusion, organ transplant, congenital infection, and oral transmission from food contaminated with insect feces [1].

Chagasic cardiomyopathy (CC) is the most serious complication of the chronic phase of CD and the major cause of morbidity and mortality among patients with CD.

The World Health Organization (WHO) considered CD as a neglected tropical disease (NTD) and a public health concern. It is one of the most prevalent imported tropical diseases in Europe, between 68,000 and 122,000 estimated cases in total among the migrant population from Latin America (LA) in 2009 although it can exist an underdiagnosis rate around 90%. Of the 3.5 million Latin Americans living in Europe [2], more than half of this migrant population, resides in Spain, followed by Italy, France and the United Kingdom. Spain is the second non-endemic country, after the United States, with the highest number of confirmed cases of CD, a global entity that affects 6–7 million people and causes about 7,000 deaths a year [3].

Prevalence differs for each group of migrants and region of origin. Depending on the type of study and population analyzed, the highest prevalence rates are found among population from Bolivia.

The presence of *T. cruzi* antibodies in maternal serum identifies mothers whose newborns are at risk of becoming infected. Since the transmission of *T. cruzi* during pregnancy cannot be prevented, early diagnosis in newborns is essential to administer appropriate etiological treatment.

## Case presentation

A 29-year-old woman from Bolivia and resident in Spain for 4 years, is admitted to the emergency room at 38 weeks of gestation due to a 3-week evolution of dyspnea, orthopnea and paroxysmal nocturnal dyspnea with sudden deterioration and dyspnea on minimal exertion.

Examination findings demonstrated oxygen saturation 96%, tachycardia and tachypnea, blood arterial pressure was 99/60 mmHg.

Urgent transthoracic echocardiogram revealed: large right ventricle dilatation with posterolateral basal aneurysm and severe systolic dysfunction; severe mitral regurgitation due to traction of the posterior leaflet; severe pulmonary hypertension (estimated pulmonary arterial systolic pressure [PASP] 75 mmHg).

Cesarean section was performed due to the diagnosis of congestive heart failure. During the procedure maternal hypotension was observed and serum therapy and phenylephrine were administered.

During cesarean section, B-Lynch technique was performed due to uterine atony. The newborn was born in good condition and no resuscitation measures were needed. The patient was admitted to the cardiac intensive care unit (ICU), where acute pulmonary edema was observed. Invasive hemodynamic monitoring of the patient was carried out with invasive ventilatory support, sedoanalgesia with remifentanil, inotropic support with dobutamine, diuretics, antibiotic prophylaxis with cefazolin, oxytocin infusion and prophylaxis gastric protection. Swan-Ganz pulmonary artery catheter was placed. Transfusion of a red blood cell concentrate was performed.

During the first hours of stay in the ICU hemodynamic stability of the patient was observed with good tolerance to inotropes, adequate diuretic response with a low dose of furosemide, and controlled uterine bleeding with hematometric stability and adequate uterine tone.

The diagnosis was dilated myocardiopathy with posterolateral aneurysm and severe left ventricle dysfunction and severe mitral regurgitation, low cardiac output and acute pulmonary edema (Forrester scale: IV) with possibility of support with intra-aortic balloon pump counterpulsation. Patient entered in heart transplantation waiting list.

The patient referred positive serology for *T. cruzi* at the beginning of the pregnancy, (positive immunoglobulin G antibodies), although she had carried out poor antenatal care. The polymerase chain reaction (PCR) requested at the ICU for *T. cruzi* was positive so benznidazole therapy was started (dosage 5 mg/kg per day) for a month and it was well tolerated. Monitoring of treatment revealed negativization of PCR for *T. cruzi*. Serology of the newborn was positive for *T. cruzi* but PCR remained negative up to 9 months after birth.

Esophagogram was normal. She was extubated and well controlled with conventional diuretic treatment. The patient was discharged due to progressive clinical improvement with cardiologic treatment after 30 days. She recovered adequate general condition so continued with medical treatment. New York heart association (NYHA) functional class: I–II.

Five years later, single-chamber implantable cardioverter defibrillator (ICD) was placed for the prevention of sudden cardiac death. A magnetic resonance imaging (MRI) revealed: severe dilatation of left ventricle, parietal thinning and inferior and inferolateral dyskinesia and transmural late enhancement. Ejection fraction of left ventricle: 28%. Slightly dilatation of left atrium and significative mitral regurgitation due to absence of posteromedial papillary muscle with normo-positioned and normal morphology of anterolateral muscle.

Three years later, NYHA class function of heart dysfunction continued I–II and Weber B classification. Severe functional mitral regurgitation was observed so percutaneous repair with Mitraclip XT device was inserted.

Currently the patient remains stable, she has a normal but self-limited life (she can go up two floors of stairs or slopes slowly to avoid breathlessness) and she continues outpatient check-ups.

## Discussion

CC is the most serious complication of the chronic phase of CD. CD has an acute phase, which, if left untreated, progresses to a chronic phase where cardiac, digestive or neurological involvement can be developed. This occurs in 30–40% of infected people 10–30 years after the acute phase and is a major cause of morbidity and mortality.

Cardiac involvement is the major cause of morbidity and mortality among patients with CD, followed by the digestive form. In fact, CD is the main cause of heart disease and death due to cardiovascular disease in a young population in Latin America. However, there are no many publications of Chagasic myocardiopathy with severe congestive heart failure during pregnancy in non-endemic areas, although CD screening protocols in pregnant women have been carried out to monitor the mother and newborn in these areas.

Cardiac disorders are developed by 20–30% of patients in the indeterminate phase. CC is usually asymptomatic until advanced stages of the disease. The transmission route, the host’s immune response, or the genetic variability of the parasite among others can determine the clinical expression of the disease. There seems to be evidence that the persistence of high parasitemia and tissue parasitism, a proinflammatory immunological profile with sustained oxidative stress and genes related to natural killer/CD8+ T-cell cytotoxicity may determinate the development of CC, although real mechanisms remain unknown to date [4].

The diagnosis of chronic CD relies on serology testing through detection of immunoglobulin G antibodies (IGA) against *T. cruzi.* The most commonly used are an enzyme-linked immunosorbent assay (ELISA), indirect immunofluorescence (IIF) and indirect hemagglutination (IHA). Parasitemia is low and intermittent in the chronic phase, thus making direct parasitological and PCR-based diagnosis methods unreliable, as PCR has low and varying sensitivities that range from 50% to 90%. The factors involved in that variability are blood volume, methodology, genes targeted, phase of infection, presence of immunosuppression, country of origin of the patient and genetic diversity of *T. cruzi*. PCR can be useful when serology tests are inconclusive, for monitoring early detection of treatment failure and in the management of immunosuppressed patients.

After the diagnosis of CD in a pregnant woman, an EKG should be performed as electrocardiographic abnormalities are frequently the first indication of CD and the presence of a typical electrocardiographic abnormality is associated with increased risk of progression to more severe CC. Pregnancy in women with heart disease is still associated with considerable morbidity and mortality rates, which strongly correlate to maternal underlying disease [5]. Therefore, patients should be evaluated for underlying cardiac disease to select appropriate management. Strict prenatal care and early risk stratification during gestation are important measures to improve the prognosis of pregnancy in women with heart disease. The newborns must be screened with PCR as IGA are not useful during the first months of life, since the IGA detected at birth in the newborn come from the mother [6].

The early manifestations of CC include weakness, palpitations, syncope, (sometimes initial manifestation), abdominal pain in the right upper quadrant of abdomen, jugular venous stasis, lower limb edema or stroke; while late manifestations are atypical chest pain, syncopal episodes, sudden cardiac death, dyspnea with exertion or at rest, orthopnea, fatigue, heart murmurs or stroke [7].

Congestive heart failure appears in advanced stages of the CC, showing the signs of systemic and pulmonary congestion. The left ventricle is frequently affected with early changes in regional motility, particularly in the inferolateral and apical region with the formation of aneurysms [8].

CC is one of the most arrhythmogenic cardiomyopathies. Electrical manifestations can precede several years to the dilation and deterioration of heart function. Sustained ventricular tachycardia is the main trigger for sudden cardiac death and the main cause of mortality [9].

Thromboembolic events are the third leading cause of death. The most common is cerebral embolism, which may be the first clinical manifestation in asymptomatic patients, followed by peripheral and pulmonary embolism. Apical aneurysm, cardiac insufficiency, and electrocardiogram (EKG) arrhythmia seem to be important risk factors in the genesis of ischemic stroke related to CD, whereas hypercoagulable states are unlikely to be major contributors to the excess stroke risk seen in CD.

Avila et al. conducted a retrospective study of 1,000 pregnant women with heart disease. 8.5% of them where women with CD. All cases of congestive heart failure were associated with dilated cardiomyopathy [10].

Etiological treatment is based in two antiparasitic drugs: benznidazole and nifurtimox. These agents are most effective in the acute phase, with cure rates of 60–80%, but treatment is recommended for all cases of reactivated infections, congenitally transmitted and for children up to 18 years old with chronic infection. There are no consensual indications por etiological treatment in the indeterminate form, however it is commonly recommended. Trypanocidal treatment did not significantly reduce cardiac clinical deterioration in patients with CC with no severe myocardial damage according to the Benznidazole Evaluation for Interrupting Trypanosomiasis (BENEFIT) trial results. Expert consensus recommends treatment of women of childbearing age to prevent congenital transmission [9].

Evidence of the usefulness of cardiac resynchronization therapy in CD is limited, although the indications for it in CP are those used for ischemia and idiopathic dilated cardiomyopathy. The efficacy of ICD in CD is not demonstrated as no large randomized clinical trials have been carried out, but European guidelines recommend using ICD in CP with and LVEF >40%. Heart transplantation is safe and effective and the indications are the same for non-Chagas patients, although infection reactivation can occur [11].

In conclusion, Chagas cardiomyopathy is a common etiology of chronic heart failure (CHF) in endemic countries but also in other countries as a result of migration. The diagnosis of CC in non-endemic areas requires a high index of suspicion and it is based on serology to detect immunoglobulin G antibodies to *T. cruzi*. Treatment of CC that causes CHF is similar to that in non-Chagasic etiology, despite the lack of strong evidence.
